# Immunoinformatics-Based Identification of B and T Cell Epitopes in RNA-Dependent RNA Polymerase of SARS-CoV-2

**DOI:** 10.3390/vaccines10101660

**Published:** 2022-10-03

**Authors:** Shabir Ahmad Mir, Mohammed Alaidarous, Bader Alshehri, Abdul Aziz Bin Dukhyil, Saeed Banawas, Yahya Madkhali, Suliman A. Alsagaby, Ayoub Al Othaim

**Affiliations:** 1 Department of Medical Laboratory Sciences, College of Applied Medical Science, Majmaah University, Al Majmaah 11952, Saudi Arabia; 2 Health and Basic Sciences Research Center, Majmaah University, Al Majmaah 11952, Saudi Arabia; 3 Department of Biomedical Sciences, Oregon State University, Corvallis, OR 97331, USA

**Keywords:** COVID-19, epitope, immunoinformatics, peptide vaccine, RNA polymerase, SARS-CoV-2

## Abstract

Introduction: The ongoing coronavirus disease 2019 (COVID-19), which emerged in December 2019, is a serious health concern throughout the world. Despite massive COVID-19 vaccination on a global scale, there is a rising need to develop more effective vaccines and drugs to curb the spread of coronavirus. Methodology: In this study, we screened the amino acid sequence of the RNA-dependent RNA polymerase (RdRp) of SARS-CoV-2 (the causative agent of COVID-19) for the identification of B and T cell epitopes using various immunoinformatic tools. These identified potent B and T cell epitopes with high antigenicity scores were linked together to design the multi-epitope vaccine construct. The physicochemical properties, overall quality, and stability of the designed vaccine construct were confirmed by suitable bioinformatic tools. Results: After proper in silico prediction and screening, we identified 3 B cell, 18 CTL, and 10 HTL epitopes from the RdRp protein sequence. The screened epitopes were non-toxic, non-allergenic, and highly antigenic in nature as revealed by appropriate servers. Molecular docking revealed stable interactions of the designed multi-epitope vaccine with human TLR3. Moreover, in silico immune simulations showed a substantial immunogenic response of the designed vaccine. Conclusions: These findings suggest that our designed multi-epitope vaccine possessing intrinsic T cell and B cell epitopes with high antigenicity scores could be considered for the ongoing development of peptide-based novel vaccines against COVID-19. However, further in vitro and in vivo studies need to be performed to confirm our in silico observations.

## 1. Introduction

In the last two decades, seven coronaviruses have infected human beings, with two main outbreaks caused by SARS-CoV (in 2002) and MERS-CoV (in 2012) [1]. In December 2019, the novel coronavirus (nCoV) emerged in Wuhan city in China, causing another global public health problem. This virus was first named as 2019-nCoV by WHO on January 12 and the associated disease was termed COVID-19 on 11 February 2020. Later, the virus causing the COVID-19 disease was identified as severe acute respiratory syndrome coronavirus-2 (SARS-CoV-2), which is a member of the Coronaviridae family of viruses [2,3]. The world is currently experiencing severe circumstances of a worldwide public health emergency due to this viral pandemic. As of 28 August 2022, over 598 million confirmed cases of COVID-19, and over 6.4 million deaths by COVID-19, have been reported globally (WHO: https://www.who.int/emergencies/diseases/novel-coronavirus-2019/situation-reports (accessed on 28 August 2022)).

A lipid envelope containing the spike proteins and the membrane proteins surrounds the positive-stranded RNA genome of SARS-CoV-2. Although coronaviruses (CoVs) are typically associated with respiratory illness, they can also cause infections in the brain and spinal cord [1,4]. The initial symptom of COVID-19 disease is fever, which can be accompanied with other symptoms such as shortness of breath, dry cough, muscle ache, headache, dizziness, sore throat, rhinorrhea, chest pain, nausea, diarrhea, and vomiting [5].

The SARS-CoV-2 binds to the host cell receptors through its spike proteins. The spike proteins also help the SARS-CoV-2 to release its viral genome into the host cell, where it is translated into two polyproteins and the structural proteins. Subsequently, the replication of the viral genome is started in the host cell [6]. Two-thirds of RNA encodes the two large non-structural polyproteins and the viral RNA-dependent RNA polymerase (RdRp) along with the associated accessory proteins. The remaining one-third of the viral genome codes for the four structural proteins (spike, membrane, envelope, and nucleocapsid) and the other helper proteins [7,8]. RdRp is derived from the polyproteins 1a and 1ab (encoded by ORF1a and ORF1ab, respectively) and is very important for the replication and transcription of the viral genome [9]. It is highly conserved among different RNA viruses. The core protein of RdRp consisting of a single chain of around 900 amino acid residues shows minimal activity. However, enhanced activity is achieved with the attachment of additional key subunits [9,10,11]. The development of effective treatment and vaccination strategies for SARS-CoV-2 is a high research priority worldwide. Various efforts are being carried out globally for the discovery of drugs and vaccines for the treatment of SARS-CoV-2 infection. Currently, 7 vaccines are approved by WHO for massive vaccination on a global scale, and over 300 additional vaccines are under development in the pre-clinical or clinical trial stage (https://www.acrobiosystems.com/A1374-An-Overview-of-Different-COVID-19-Vaccines.html?gclid=EAIaIQobChMIn__dp5em-AIV2hXUAR2XOgJPEAAYAyAAEgKBCPD_BwE (accessed on 28 August 2022)).

The conventional methods for designing a vaccine (involving whole organisms or large proteins) lead to excessive antigenic load in addition to increasing the chances of allergic responses and toxicity [12]. This problematic concern can be overcome by designing peptide-based vaccines that encompass short immunogenic peptides with the ability to elicit strong and targeted immune responses, thereby avoiding the chances of allergenic and toxic reactions [12]. There are several recent studies available in the literature where the immunoinformatics-based vaccine designing efforts have been employed to address the challenge of vaccine construction for SARS-CoV-2 [13,14,15,16,17,18,19,20,21,22,23,24,25]. To add up to the available literature for developing an efficient peptide-based vaccine for SARS-CoV-2, here, we applied the in silico approach to identify the immunodominant B and T cell epitopes of the RdRp protein and develop a multi-epitope vaccine against SARS-CoV-2.

## 2. Methodology

### 2.1. Protein Sequence Retrieval and Determination of Antigenicity

The primary amino acid sequence of the RdRp protein of SARS-CoV-2 (accession number; YP_009725307.1) was retrieved from the NCBI database (https://www.ncbi.nlm.nih.gov/protein (accessed on 25 October 2021)) and its antigenicity was checked by the ANTIGENpro tool of the Scratch protein predictor (http://scratch.proteomics.ics.uci.edu/ (accessed on 25 October 2021)).

### 2.2. B Cell Epitope Prediction

We used the B cell epitope prediction server, BCPREDS server 1.1 (http://ailab-projects1.ist.psu.edu:8080/bcpred/predict.html (accessed on 10 November 2021)), with default settings of various parameters, to identify the linear B cell epitopes of the RdRp protein. The VaxiJen v2.0 server (http://www.ddg-pharmfac.net/vaxijen/VaxiJen/VaxiJen.html (accessed on 11 November 2021)) was used to evaluate the antigenicity of the predicted epitopes [26]. The epitopes with antigenicity scores above 0.4 were selected for further investigation.

### 2.3. Prediction of Helper T Lymphocyte (HTL) Epitopes and Their Screening

The IEDB server (http://tools.iedb.org/mhcii/ (accessed on 15 November 2021)) was used for the prediction of MHCII binding/HTL epitopes in the protein sequence of RdRp [27]. Based on their percentile rank, the top 40 HTL epitopes were sorted. Epitopes with the lower percentile rank were considered to have higher MHCII binding affinity [28]. The selected epitopes were verified for their antigenicity by Vaxijen v2.0 [26] using the default threshold value of 0.4 for the prediction. The epitopes with an antigenicity score above 0.4 were screened for in silico IFN-γ induction using the IFNepitope server.

The high-scoring epitopes (antigenicity score > 0.4) with the ability to induce IFN-γ and cell-mediated immunity were identified by the IFNepitope server (http://crdd.osdd.net/raghava/ifnepitope/ (accessed on 16 November 2021)). The prediction was made by a motif and support vector machine (SVM) hybrid approach [29]. Only the positive epitopes able to induce interferon-γ (IFN-γ) were selected for further experiments.

### 2.4. Prediction of Cytotoxic T Lymphocyte (CTL) Epitopes

MHCI binding/CTL epitopes for supertypes A2, A3, and B7 were predicted in the RdRp sequence using an online NetCTL 1.2 server (http://www.cbs.dtu.dk/services/NetCTL/ (accessed on 25 November 2021)) [30]. The epitopes were predicted by choosing default parameters for the three MHCI supertypes: A2, A3, and B7. The A2, A3, and B7 supertypes were selected because these three together cover >88% of the global inhabitants [27,30,31]. The default settings of NetCTL 1.2 (epitope identification threshold of 0.75, weight on TAP transport efficiency of 0.05, and weight on C terminal cleavage of 0.15) were chosen for epitope prediction. Here again, the VaxiJen v2.0 server was used to evaluate the antigenicity of the predicted epitopes. The CTL epitopes with an antigenicity score above 0.4 were selected for further investigation.

### 2.5. Toxicity Assessment and Prediction of the Allergenicity of the Screened Epitopes

The toxicity and allergenicity of all the screened HTL, CTL, and B cell epitopes were assessed by employing the ToxinPred server (https://webs.iiitd.edu.in/raghava/toxinpred/pep_test.php (accessed on 27 November 2021)) [27,32] and AllerTOP v2.0 server (http://www.ddg-pharmfac.net/AllerTOP (accessed on 28 November 2021)) [33], respectively. The default settings of both the servers were used for the respective predictions. The non-toxic and non-allergic HTL, CTL, and B cell epitopes with antigenicity scores more than 0.4 were selected for vaccine construction.

### 2.6. Construction of Multi-Epitope-Based Vaccine

The screened CTL, HTL, and B cell epitopes from the target protein were joined together through linkers (short and specific amino acid sequences) to generate the multi-epitope vaccine sequence. The different linkers used in this study include the B epitope linker (KK), HTL linker (GPGPG), and CTL linker (AAY). β-defensin 1 (Uniprot Id: P60022) was used as an adjuvant and linked through an EAAAK linker at the N-terminus of the vaccine sequence [34].

### 2.7. Prediction of Antigenicity and Allergenicity of the Designed Vaccine Candidate

The servers VaxiJen v2.0 (http://www.ddg-pharmfac.net/vaxijen/VaxiJen/VaxiJen.html (accessed on 10 December 2021)) and ANTIGENpro (http://scratch.proteomics.ics.uci.edu/ (accessed on 11 December 2021)) were applied for the antigenicity evaluation of the designed multi-epitope vaccine construct [26,35]. The VaxiJen v2.0 server predicts the antigenicity on the basis of physicochemical properties of the input protein and the ANTIGENpro server predicts the antigenicity on the basis of protein microarray data analysis.

The server AllerTOP v2.0 (http://www.ddg-pharmfac.net/AllerTOP (accessed on 12 December 2021)) and AllergenFP (https://ddg-pharmfac.net/AllergenFP/feedback.py (accessed on 14 December 2021)) were used to predict the allergenicity of the designed multi-epitopic vaccine [33,36]. AllerTOP v2.0 uses machine learning methods for the classification of allergens whereas AllergenFP uses SVM modules to predict the allergenicity of proteins.

### 2.8. Physiochemical Properties and Secondary Structure Prediction of the Constructed Vaccine

The ProtParam server (http://web.expasy.org/protparam/ (accessed on 15 December 2021)) was used to predict the physicochemical properties of the designed vaccine construct [37] and PSIPRED server (http://bioinf.cs.ucl.ac.uk/psipred/ (accessed on 16 December 2021)) [38] was used to predict its secondary structure [39].

### 2.9. Disorder Profile Generation

The disorder profile of the designed vaccine was produced through the DisProt server (http://original.disprot.org/metapredictor.php (accessed on 20 December 2021)) using the PONDR pool of four predictors [40,41,42] and IUPRED 2A predictor (https://iupred2a.elte.hu/ (accessed on 20 December 2021)) [43,44,45,46].

### 2.10. Tertiary Structure Prediction and Validation

The tertiary structural model of the designed vaccine construct was generated using the I-TASSER web server (https://zhanglab.ccmb.med.umich.edu/I-TASSER/ (accessed on 21 December 2021)) [47], which provides energy minimized models through iterative fragment assembly simulations, which are template-based. The topmost model was selected and evaluated for validation using ERRAT (https://servicesn.mbi.ucla.edu/ERRAT/ (accessed on 30 December 2021)) and PROCHECK (https://servicesn.mbi.ucla.edu/PROCHECK/ (accessed on 30 December 2021)) [48] web servers.

### 2.11. Molecular Interaction of the Designed Multi-Epitope Vaccine with TLR-3

The possibility of interaction between the immune receptor molecule (human TLR3) and the designed vaccine model was predicted by molecular docking experiments. The human TLR-3 protein structure (PDB ID: 1ZIW) was downloaded from the PDB database and the vaccine structure (predicted and evaluated models) was used as the ligand for this receptor. The interaction between the two was performed using the GRAMM-X simulation web server (http://vakser.compbio.ku.edu/resources/gramm/grammx/ (accessed on 31 December 2021)) [49,50] and interactions were visualized using PyMOL software [https://pymol.org/2/ (accessed on 2 January 2022)] [51].

### 2.12. In Silico Immune Stimulation Assay

To evaluate the immunological responses of the designed vaccine, its in silico immune simulation analysis was performed using C-ImmSim 10.1 server (http://150.146.2.1/C-IMMSIM/index.php?page=0 (accessed on 5 January 2022)). The simulation was performed with default parameters, and we evaluated the immune stimulation for only one injection of the vaccine [52].

## 3. Results

### 3.1. B-Cell Epitopes

The RdRp protein is a probable antigen with a predicted antigenicity score of 0.620516 as revealed by the ANTIGENpro tool. The linear B cell epitopes of RdRp protein were predicted using BCPREDS server 1.1. The epitopes chosen on the basis of prediction scores were screened for their antigenicity using VaxiJen v2.0 server (Supplementary Table S1) and those with an antigen score of more than 0.4 were selected for further analysis. The BCPREDS server 1.1 analysis of the RdRp protein sequence revealed 13 B-cell epitopes among which only 5 epitopes possessed the antigenicity as revealed by the VaxiJen v2.0 server. Among these, three potential non-allergic, non-toxic, and non-overlapping epitopes with high antigenicity scores (>0.4) were selected for final vaccine construction (Table 1).

### 3.2. HTL Epitopes

The HTL epitopes of RdRp protein were predicted using the IEDB server for human MHC II alleles. The antigenic protein sequence of RdRp was analyzed on the T cell epitope-MHC II binding tool of IEDB while choosing the entire human HLA reference set and keeping other parameters of the server in the default setting. The rationale behind choosing the entire HLA reference set was that this would produce epitopes covering almost all the globally present MHC II alleles [27,28]. Based on their least percentile rank revealing their high affinity, the top 40 epitopes were selected and among these, 20 were antigenic as revealed by Vaxijen v2.0 server analysis (Supplementary Table S2). These 20 epitopes were further assessed for their IFN-γ-inducing potential and only 15 epitopes were positive for IFN-γ induction. All the 15 IFN-γ-inducing epitopes were non-toxic, whereas one out of the 15 IFN-γ-inducing epitopes proved to be a probable allergen. The final selection of epitopes was made on the basis of overlapping sequences and 10 epitopes with positive IFN-γ-inducing potential and possessing high antigenicity scores (>0.4) were finally selected for vaccine construction (Table 2).

### 3.3. CTL Epitopes

The CTL epitopes for supertypes A2, A3, and B7 were predicted using the NetCTL 1.2 server. A total of 35, 48, and 25 epitopes (for the supertypes A2, A3, and B7, respectively) were predicted in the RdRp protein sequence (Supplementary Table S3). Out of these, only 16, 28, and 13 were antigenic as revealed by the Vaxijen v2.0 server-based antigenicity analysis. All these epitopes were non-toxic in nature as analyzed by the ToxinPred server (Supplementary Table S4). Further selection of the epitopes was carried out based on their allergenicity. The server AllerTOP v2.0 ascertained only 18 CTL epitopes (5 for A2, 8 for A3, and 5 for B7) as probable non-allergens and hence good antigens for further experiments (Table 3).

### 3.4. Multi-Epitope Vaccine Design

The selected antigenic peptides/epitopes were joined together through the linkers to create the multi-epitope vaccine candidate. In addition to maintaining the construct immunogenicity, the linkers also make the resulting protein complex more stable and flexible [27,28]. Our designed vaccine construct comprised 18 CTL epitopes, 10 HTL epitopes, and 3 B cell epitopes (Supplementary Figure S1). Additionally, for improving the immunogenicity of the multi-epitope vaccine, we tagged the construct with an adjuvant (β-defensin) at the N terminus.

### 3.5. Prediction of the Antigenicity and Allergenicity of the Constructed Vaccine

One of the characteristics of an ideal vaccine is that the vaccine must be antigenic and non-allergic in nature. Our designed vaccine was observed to be antigenic with antigenicity score of 0.7006 as predicted by the VaxiJen v2.0 server. The construct was also identified as a probable non-allergen by the AllerTOP v2.0 server.

### 3.6. Physiochemical Characterization of the Designed Vaccine

As revealed by ProtParam server analysis, the designed vaccine construct with 540 amino acids has a predicted molecular weight of 58.245 kDa. It is basic in nature (theoretical pI 9.93) and would possess a half-life of 30 h in mammalian reticulocytes (in vitro) and >10 h in *Escherichia coli* (in vivo). Its predicted instability index of 21.73 classifies the protein as stable. The designed vaccine is thermostable as revealed by its aliphatic index (88.17) and its average hydropathicity is 0.034.

### 3.7. Prediction of Secondary Structure and Disordered Residues

**Figure 1:**
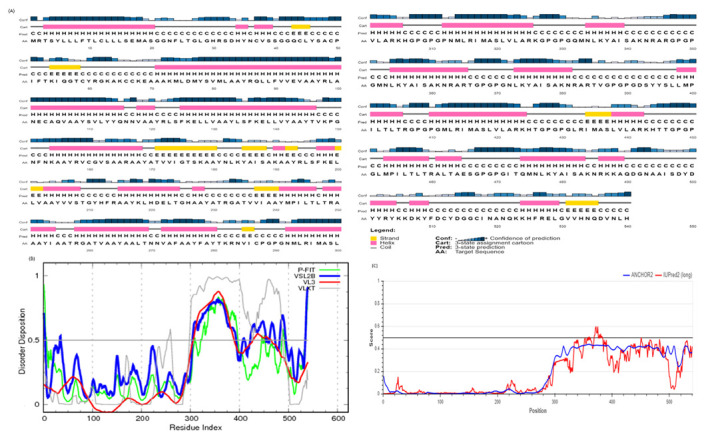
(**A**) Secondary structure prediction of the designed vaccine construct (graphical result) as revealed by the PSIPRED server. The secondary structures are indicated by different colors: α-helix (shown in pink), β-sheet (shown in yellow), and coil (shown in grey). (**B**) Intrinsic disorder in residues of the designed vaccine construct as predicted by PONDR^®^ pool of predictors (PONDR-FIT, PONDR-VSL2B, PONDR-VL3, PONDR-VLXT). (C) Disorder in residues of the designed vaccine construct as predicted by the IUPRED 2A server.

The secondary structure of the designed vaccine construct as predicted by the PESIPRED 4.0 server contains several α-helices, few β-strands, and many coils (Figure 1). Its disorder profiles, as generated through the DisProt server and IUPRED 2A predictor, are also shown in Figure 1.

### 3.8. Prediction and Quality Assurance of the Tertiary Structure of the Designed Vaccine

**Figure 2:**
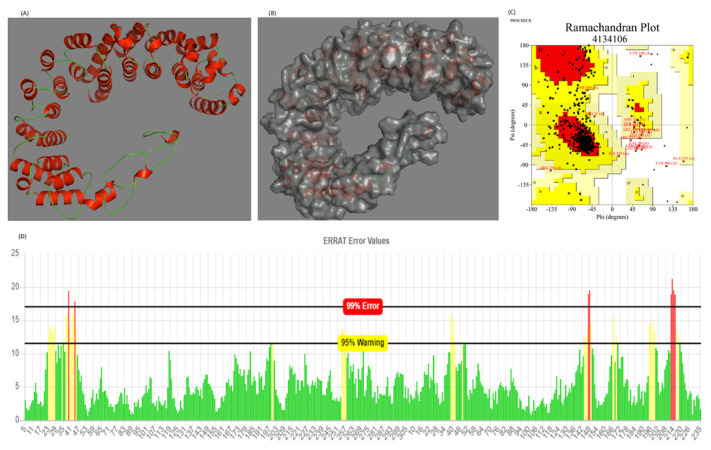
(**A**,**B**) Structure of the selected 3D model of the final vaccine construct (cartoon and surface representation, respectively). Red and green represent the helical and loop region, respectively. (**C**) Ramachandran plot of the predicted model of the designed vaccine using the PROCHECK server (76.3%, 19.6%, 2.6%, and 1.5 in the favored, allowed, outlier, and disallowed regions, respectively). (**D**) Validation of the overall quality of the predicted model on ERRAT2 analysis (overall quality factor = 91.149).

The designed 540-residue-long vaccine construct was modeled on the I-TASSER web server. The server revealed the five top models with C-scores ranging from −1.01 to −3.77, among which the topmost model with the highest C-score (−1.01) was selected for further analysis. A C-score with a higher value indicates a model with a greater confidence and vice versa. The selected model majorly comprised alpha helices and loops, with almost no contribution from beta sheets (Figure 2). To validate the structural quality of the predicted model, PROCHECK and ERRAT analyses were performed. The PROCHECK generated a Ramachandran plot of the 3D model of the vaccine, showing that 98.5% of the residues were localized in the favored regions and only 1.5% of them were positioned in the disallowed regions, which confirms the overall quality of the vaccine model (Figure 2). Ideally, a reliable model has at least 90% of its residues in the favored region [53]. The overall quality factor of the designed vaccine model as revealed by ERRAT analysis was 91.149, which signifies a good-quality model worth investigating further (Figure 2). An ERRAT score greater than 50 denotes a good-quality model [54].

### 3.9. Molecular Docking of the Vaccine Construct with TLR-3

**Figure 3:**
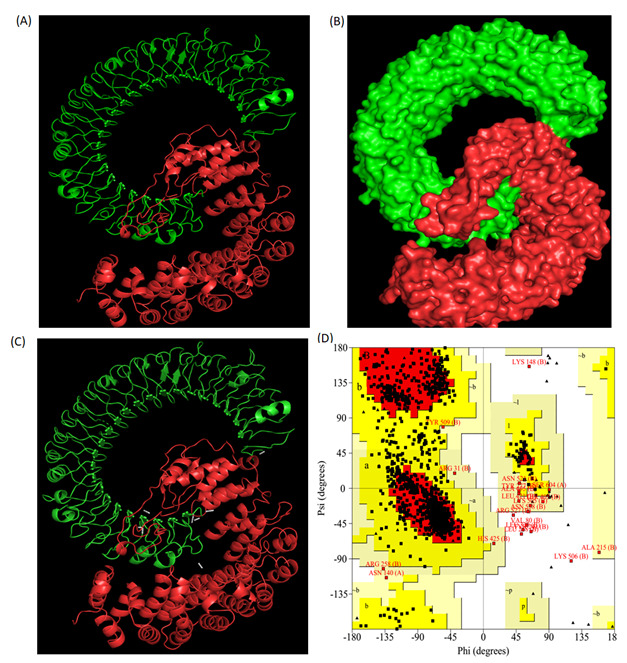
(**A**) Cartoon representation and (**B**) surface view of the structure and interactions of the docking complex (TLR3-designed vaccine). TLR3 is shown in green whereas designed vaccine is shown in red. (**C**) The dotted lines in white represent the polar interactions between the TLR3 and designed vaccine. (**D**) Ramachandran plot of the docking complex as revealed by PROCHECK server analysis.

An appropriate interaction between the antigen molecules and the immune receptor is essential for generating an immune response. Therefore, to ensure that the designed vaccine construct is capable of interacting with the immune receptor TLR-3, we executed a docking study using the GRAMM-X simulation web server. This docking server is exclusively designed for docking pairs of protein molecules. The protein-protein docking model complex produced by the GRAMM-X simulation web server and visualized by PyMOL is shown in Figure 3. The interactions between TLR-3 protein (chain A) and the designed vaccine construct (chain B) were also visualized by PyMOL software (see Figure 3). The PROCHECK-generated Ramachandran plot analysis of the protein-protein docking model complex showed that 99.3% of the residues were localized in the favored regions whereas only 0.7% of them were positioned in the disallowed regions, which verifies the overall quality of the docking complex (Figure 3). Further, the molecular dynamics simulations of the docked complex were performed on the CABS-flex server (http://biocomp.chem.uw.edu.pl/CABSflex/ (accessed on 8 January 2022)). The root-mean-square fluctuation (RMSF) of the amino acid residues of both the chains of the complex (chain A, TLR3; chain B, designed vaccine construct) are shown in Supplementary Figure S2.

### 3.10. In Silico Immunogenicity of the Designed Vaccine

**Figure 4:**
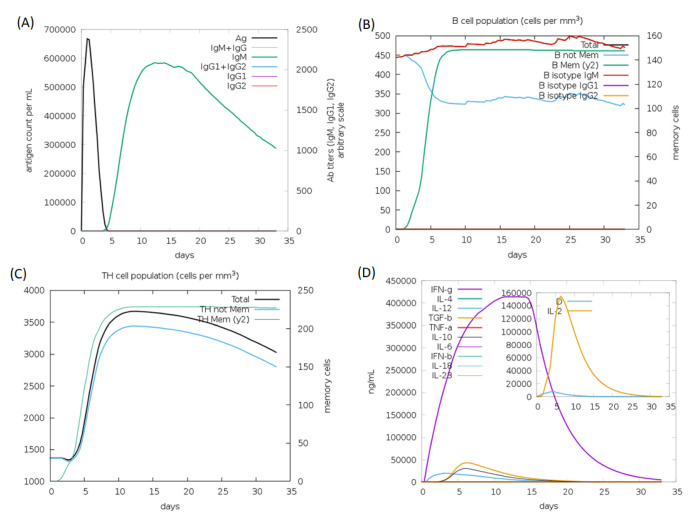
C-ImmSim server prediction results of the immune response against the designed multi-epitope vaccine; (**A**) Antigen and immunoglobulins; (**B**) B lymphocyte population; (**C**) Helper T cell population; (**D**) Induced levels of interleukins and cytokines.

The in silico host immune responses to our designed multi-epitope vaccine are shown in Figure 4. The immune responses after in silico stimulation included high IgM levels and increased lymphocyte populations. Additionally, robust cytokine and interleukin responses were observed.

## 4. Discussion

SARS-CoV-2 has infected millions of people worldwide and is now considered a public health emergency. The development of efficient vaccines and drugs against COVID-19 disease is urgently needed to get rid of this pandemic. Several efforts are being carried out globally for the development of efficient and safe vaccines for this disease. Bioinformatics-based approaches offer a novel in silico methodology for the identification of potential T and B cell epitopes in the viral protein sequences. These epitopes are useful for the development of effective multi-epitope-based vaccines. Currently, several vaccine design efforts have been reported in the literature to address the challenge of vaccine construction for SARS-CoV-2 [13,14,15,16,17,18,19,20,21,22,23,24,25]. In most of the available studies, the immunoinformatics-based protocols were applied for the prediction of important B and T cell epitopes in various structural and non-structural proteins of SARS-CoV-2. The predicted epitopes were screened, and the selected ones were joined together to develop a multi-epitope vaccine. Here, we employed the immunoinformatic approach for the prediction of potential B and T cell epitopes in the RdRp protein of SARS-CoV-2. After scanning the primary amino acid sequence of RdRp protein using different high efficiency immunoinformatics tools, various B and T cell epitopes were identified. After proper screening of these epitopes using suitable bioinformatics servers, we identified and selected 3 B cell epitopes (Table 1), 10 HTL epitopes (Table 2), and 18 CTL epitopes (Table 3), which are highly antigenic and do not possess any toxicity or allergenicity (see Supplementary Materials). All the selected HTL epitopes are potential IFN-γ inducers as revealed by the IFNepitope server. These selected peptide epitopes need further investigation under in vitro and in vivo conditions to confirm our in silico observations. Moreover, as reported in the literature, different peptide epitopes capable of activating several HLA restricted specific T cells can be combined with B cell epitopes to form a common vaccine formulation. Epitope-based vaccines are safe and have been reported to induce more potent immune responses than the parallel whole protein vaccines [55]. Accordingly, we joined our screened and selected B and T cell epitomes through different linkers to make the multi-epitope vaccine construct (Supplementary Figure S1). The multi-epitope vaccines are easy to produce in bulk using the codon optimization and gene cloning techniques. Since peptide vaccines are known to produce low immune responses in the absence of an efficient adjuvant, we linked the TLR-3 agonist β-defensin as an adjuvant to the multi-epitope vaccine construct. We reported here some additional in silico analysis, including the characterization and modeling of the vaccine construct and the validation of the generated vaccine model. Further, the molecular docking of the designed vaccine model with the TLR-3 structural model and the desired interaction between the two was also analyzed and reported (Figure 3). Moreover, the in silico host immune responses to our designed multi-epitope vaccine were observed to be quite significant (Figure 4). Our results suggest that this vaccine construct containing the immunogenic B and T cell epitopes of RdRp can be considered for the ongoing development of peptide-based vaccines against COVID-19. However, further in vitro and in vivo investigations are required to ensure the real potential of our designed multi-epitope vaccine to combat COVID-19.

## 5. Conclusions

In this study, the B and T cell epitopes in RNA-dependent RNA polymerase of SARS-CoV-2 were identified and screened using different web-based bioinformatics servers. The selected non-allergic and non-toxic epitopes with high antigenicity scores were joined together through linkers to make the multi-epitope vaccine construct. The vaccine construct was modeled, and the 3D model was docked with TLR3 using efficient docking servers. Stable molecular interaction between the vaccine model and TLR3 was observed in the docking complex. Further, the docking complex was validated by the PROCHEK server, and the favorable Ramachandran plot of the complex confirmed its stability and flexibility.

## Figures and Tables

**Table 1:** Non-allergic and non-toxic antigenic B cell epitopes with respective antigenic scores.

S No	Starting Amino Acid Position	Peptide Epitope	Bcpreds Server Score	Antigenicity Score (On Vaxijen V2.0)
1	443	AQDGNAAISDYDYYRY	0.987	0.5716
2	477	DKYFDCYDGGCINANQ	0.973	0.6443
3	347	HFRELGVVHNQDVNLH	0.938	1.4181

**Table 2:** Non-allergic and non-toxic antigenic HTL epitopes (with respective antigenic score and percentile rank) selected for constructing the multi-epitope vaccine. All these epitopes were positive for in silico IFN-γ induction (run on the IFNepitope server).

S No.	Peptide Epitope	Starting Amino Acid Position	Percentile Rank	Antigenicity Score (On Vaxijen V2.0)
1	NMLRIMASLVLARKH	208	0.01	0.4897
2	PNMLRIMASLVLARK	207	0.01	0.4128
3	QMNLKYAISAKNRAR	121	0.07	1.5044
4	MNLKYAISAKNRART	122	0.08	1.4377
5	NLKYAISAKNRARTV	123	0.15	1.3422
6	DSYYSLLMPILTLTR	25	0.16	0.5134
7	MLRIMASLVLARKHT	209	0.22	0.5283
8	LRIMASLVLARKHTT	210	0.32	0.6646
9	LMPILTLTRALTAES	31	0.37	0.4225
10	ITQMNLKYAISAKNR	119	0.41	1.5061

**Table 3:** Non-allergic and non-toxic antigenic CTL epitopes (with respective antigenic scores) selected for designing the multi-epitope vaccine construct.

S No	Start	Peptide Epitope	Supertype	Antigenicity Score (On Vaxijen V2.0)
1	899	MLDMYSVML	A2	0.5626
2	467	RQLLFVVEV	A2	0.816
3	654	RLANECAQV	A2	0.9246
4	784	SVLYYQNNV	A2	0.5524
5	365	RLSFKELLV	A2	0.864
6	366	LSFKELLVY	A3	0.723
7	409	TVKPGNFNK	A3	1.378
8	10	RVCGVSAAR	A3	0.608
9	585	ATVVIGTSK	A3	0.757
10	543	NLKYAISAK	A3	1.351
11	365	RLSFKELLV	A3	0.864
12	341	VVSTGYHFR	A3	1.474
13	890	KLHDELTGH	A3	0.4147
14	581	ATRGATVVI	B7	0.569
15	242	MPILTLTRA	B7	0.806
16	579	IAATRGATV	B7	0.8883
17	399	AALTNNVAF	B7	0.638
18	528	FAYTKRNVI	B7	1.0296

## Data Availability

Not applicable.

## Supplementary Materials

The following supporting information can be downloaded at: https://www.mdpi.com/article/10.3390/vaccines10101660/s1, Table S1. B-cell epitopes predicted using the BCPREDS server along with their VaxiJen score. Table S2. Top 40 HTL epitopes of RdRp predicted using IEDB server with their VaxiJen score. Table S3. CTL epitopes predicted using NetCTL 1.2 server with their VaxiJen score and ToxinPred server report. Table S4. ToxinPred report for CTL epitopes having VaxiJen score above the threshold of 0.4. Figure S1. The designed vaccine construct sequence. The sequence of epitopes in the designed vaccine is: Adjuvant-CTL epitopes-HTL epitopes-B cell epitopes. The first 68 amino acids are occupied by the adjuvant, β-defensin 1 (Bue color), next 5 amino acids are of EAAAK linker ((Orange color) and the amino acids from 74–286 constitute the CTL epitopes (Red color) whereas the amino acids from 287–486 and from 487–540 correspond to HTL (Green color) and B-cell epitopes (Black color), respectively. All the linkers, except EAAAK are represented in purple color. Figure S2. Molecular dynamics simulations of the docked complex (TLR3-Designed vaccine). (A) The root-mean-square fluctuation (RMSF) values of the amino acid residues of Chain A (TLR3) of the docked complex. (B) RMSF values of the amino acid residues of Chain B (designed vaccine) of the docked complex.

## Abbreviations

CTL: Cytotoxic T-lymphocytes; HTL: Helper T-lymphocytes; IEDB: Immune Epitope Database; IFN-γ: Interferon gamma; MHC: Major histocompatibility complex; MERS: Middle East Respiratory Syndrome; SARS: Severe Acute Respiratory Syndrome; TLR3: Toll-like receptor 3; WHO: World Health Organization.
