# Supplementary material for: Immunoinformatics-Based Identification of B and T Cell Epitopes in RNA-Dependent RNA Polymerase of SARS-CoV-2
Source: Vaccines (Basel). 2022 Oct 3;10(10):1660. doi: 10.3390/vaccines10101660 (PMC9611076; doi:10.3390/vaccines10101660)
Supplement: Supplementary file 1 [file vaccines-10-01660-s001.zip › vaccines-1931722-supplementary.pdf]

Title: Immunoinformatics based identification of B and T-Cell epitopes in RNA dependent RNA polymerase of SARS-CoV-2

Shabir Ahmad Mir [1]*, Mohammed Alaidarous [1, 2], Bader Alshehri [1], Abdul Aziz Bin Dukhyil [1], Saeed Banawas [1, 2, 3], Yahya Madkhali [1], Suliman A Alsagaby[1], Ayoub Al Othaim [1]

[1] Department of Medical Laboratory Sciences, College of Applied Medical Science, Majmaah University, Al Majmaah-11952, Saudi Arabia

[2] Health and Basic Sciences Research Center, Majmaah University, Al Majmaah 11952, Saudi Arabia

[3] Department of Biomedical Sciences, Oregon State University, Corvallis, OR 97331, USA

*** Address for correspondence**

Dr Shabir Ahmad Mir

Assistant Professor

Department of Medical Laboratory Sciences,

College of Applied Medical Science,

Majmaah University, Saudi Arabia.

Phone: +966536300645

Email: s.mir@mu.edu.sa

Supplementary Table S1. B-cell epitopes predicted using the BCPREDS server along with their VaxiJen score.

S No	Starting amino acid Position	Amino acid sequence of the Peptide epitope	BCPREDS server Score	VaxiJen score
**1**	219	FGDFIQTTPGSGVPVV	0.996	0.3090
**2**	908	TNDNTSRYWEPEFYEA	0.994	0.0979
**3**	677	PGGTSSGDATTAYANS	0.989	0.2651
**4**	443	AQDGNAAISDYDYYRY	0.987	0.5716
**5**	20	TPCGTGTSTDVVYRAF	0.986	0.3946
**6**	794	MSEAKCWTETDLTKGP	0.981	0.5943
**7**	477	DKYFDCYDGGCINANQ	0.973	0.6443
**8**	821	KQGDDYVYLPYPDPSR	0.973	0.0896
**9**	866	AYPLTKHPNQEYADVF	0.973	0.1365
**10**	283	FDRYFKYWDQTYHPNC	0.959	0.3082
**11**	135	DEGNCDTLKEILVTYN	0.942	-0.1097
**12**	347	HFRELGVVHNQDVNLH	0.938	1.4181
**13**	48	FLKTNCCRFQEKDEDD	0.919	0.5726

Supplementary Table S2. Top 40 HTL epitopes of RdRP predicted using IEDB server with their VaxiJen score.

S No	Amino acid (AA) sequence of the Peptide epitope	HLA allele to which epitope can bind	Starting AA Position	Percentile Rank	Vaxijen Score
1	AMPNMLRIMASLVLA	HLA-DRB1*15:01	205	0.01	0.096
2	MPNMLRIMASLVLAR	HLA-DRB1*15:01	206	0.01	0.1001
3	NMLRIMASLVLARKH	HLA-DRB1*15:01	208	0.01	0.4897
4	PNMLRIMASLVLARK	HLA-DRB1*15:01	207	0.01	0.4128
5	RAMPNMLRIMASLVL	HLA-DRB1*15:01	204	0.01	0.0255
6	NEFYAYLRKHFSMMI	HLA-DRB1*11:01	323	0.02	0.2279
7	EFYAYLRKHFSMMIL	HLA-DRB1*11:01	324	0.05	0.39
8	VNEFYAYLRKHFSMM	HLA-DRB1*11:01	322	0.05	0.1645
9	QMNLKYAISAKNRAR	HLA-DPA1*02:01/DPB1*14:01	121	0.07	1.5044
10	MNLKYAISAKNRART	HLA-DPA1*02:01/DPB1*14:01	122	0.08	1.4377
11	FVNEFYAYLRKHFSM	HLA-DRB1*11:01	321	0.11	0.0518
12	TQMNLKYAISAKNRA	HLA-DPA1*02:01/DPB1*14:01	120	0.11	1.4529
13	QKLLKSIAATRGATV	HLA-DPA1*02:01/DPB1*14:01	153	0.12	0.0888
14	NMLRIMASLVLARKH	HLA-DPA1*02:01/DPB1*14:01	208	0.13	0.4897
15	PNMLRIMASLVLARK	HLA-DPA1*02:01/DPB1*14:01	207	0.14	0.4128
16	HQKLLKSIAATRGAT	HLA-DPA1*02:01/DPB1*14:01	152	0.15	0.0799
17	KLLKSIAATRGATVV	HLA-DPA1*02:01/DPB1*14:01	154	0.15	0.1825
18	NLKYAISAKNRARTV	HLA-DPA1*02:01/DPB1*14:01	123	0.15	1.3422
19	DSYYSLLMPILTLTR	HLA-DRB1*01:01	25	0.16	0.5134
20	FHQKLLKSIAATRGA	HLA-DPA1*02:01/DPB1*14:01	151	0.16	0.2173
21	SYYSLLMPILTLTRA	HLA-DRB1*01:01	26	0.16	0.5509
22	VDSYYSLLMPILTLT	HLA-DRB1*01:01	24	0.16	0.5149
23	VVDSYYSLLMPILTL	HLA-DRB1*01:01	23	0.16	0.4474
24	KELLVYAADPAMHAA	HLA-DRB3*01:01	159	0.2	0.1356
25	MLRIMASLVLARKHT	HLA-DPA1*02:01/DPB1*14:01	209	0.2	0.5283
26	DFVNEFYAYLRKHFS	HLA-DRB1*11:01	320	0.22	0.0385
27	MLRIMASLVLARKHT	HLA-DRB1*15:01	209	0.22	0.5283
28	PVVDSYYSLLMPILT	HLA-DRB1*01:01	22	0.24	0.4117
29	NFNVLFSTVFPPTSF	HLA-DPA1*01:03/DPB1*04:01	102	0.3	0.5021
30	FKELLVYAADPAMHA	HLA-DRB3*01:01	158	0.31	0.0669
31	PNMLRIMASLVLARK	HLA-DRB1*01:01	207	0.31	0.4128
32	LRIMASLVLARKHTT	HLA-DPA1*02:01/DPB1*14:01	210	0.32	0.6646
33	MPNMLRIMASLVLAR	HLA-DPA1*02:01/DPB1*14:01	206	0.34	0.1001
34	LMPILTLTRALTAES	HLA-DRB1*08:02	31	0.37	0.4225
35	VFHLYLQYIRKLHDE	HLA-DRB4*01:01	460	0.37	0.2814
36	DVFHLYLQYIRKLHD	HLA-DRB4*01:01	459	0.38	0.3189
37	MPILTLTRALTAESH	HLA-DRB1*08:02	32	0.38	0.3258
38	FHLYLQYIRKLHDEL	HLA-DRB4*01:01	461	0.39	0.3059
39	ITQMNLKYAISAKNR	HLA-DPA1*02:01/DPB1*14:01	119	0.41	1.5061
40	MPNMLRIMASLVLAR	HLA-DRB1*01:01	206	0.41	0.1001

Supplementary Table S3. CTL epitopes predicted using NetCTL 1.2 server with their VaxiJen score and ToxinPred server report.

A2 supertype binding peptides	Start	VaxiJen score	A3 supertype binding peptides	Start	VaxiJen score	B7 supertype binding peptides	Start	VaxiJen score
**TMADLVYAL**	123	0.8208	RLYYDSMSY	513	-0.1146	FPPTSFGPL	321	1.6296
**LMIERFVSL**	854	-0.0605	HLYLQYIRK	882	0.492	VPHISRQRL	111	0.1685
**FVNEFYAYL**	741	-0.4788	KLFDRYFKY	281	-0.0533	KPYIKWDLL	263	0.4825
**NLIDSYFVV**	64	0.4282	RQFHQKLLK	569	-1.0437	AVTANVNAL	699	0.3733
**ILHCANFNV**	307	0.5385	KSAGFPFNK	500	1.1874	KARLYYDSM	511	-0.0551
**MLDMYSVML**	899	0.5626	TSFGPLVRK	324	1.7142	MPNMLRIMA	626	-0.2116
**NMLRIMASL**	628	-0.3326	MTNRQFHQK	566	0.5341	YVRNLQHRL	719	-0.4066
**YTMADLVYA**	122	1.0142	MVMCGGSLY	666	-0.259	IPTITQMNL	536	0.7997
**FVDGVPFVV**	334	0.1285	HISRQRLTK	113	-0.8467	ATRGATVVI	581	0.5688
**LLMPILTLT**	240	0.5803	IMASLVLAR	632	0.5587	TPCGTGTST	20	0.7838
**SLSHRFYRL**	647	0.4779	AVAKHDFFK	95	-0.8254	LPYPDPSRI	829	-0.1931
**SLAIDAYPL**	861	0.7576	LVASIKNFK	775	0.8148	CPAVAKHDF	93	1.1025
**RQLLFVVEV**	467	0.816	HLMGWDYPK	613	-0.2495	YLRKHFSMM	748	0.4887
**NLLKDCPAV**	88	-0.5347	LIDSYFVVK	65	0.4939	RVRQALLKT	181	-0.5069
**SVFNICQAV**	692	-0.416	RVYANLGER	173	0.6654	MPILTLTRA	242	0.8061
**MILSDDAVV**	756	-0.1842	KNFKSVLYY	780	0.1643	IAATRGATV	579	0.8883
**SLLMPILTL**	239	0.2133	MASLVLARK	633	1.3011	AALTNNVAF	399	0.6381
**VMCGGSLYV**	667	-0.2332	CSQHTMLVK	813	0.1398	RAMPNMLRI	624	0.0566
**RLANECAQV**	654	0.9246	LSFKELLVY	366	0.7234	KPGNFNKDF	411	0.8932
**KQGDDYVYL**	821	0.4041	TVKPGNFNK	409	1.3778	GPLVRKIFV	327	-0.0569
**QLLFVVEVV**	468	0.821	AIDAYPLTK	863	0.5972	SAKNRARTV	549	0.9661
**RIMASLVLA**	631	0.1241	FAVSKGFFK	422	-0.2311	SIKNFKSVL	778	0.2414
**SVLYYQNNV**	784	0.5524	CSLSHRFYR	646	0.629	FAYTKRNVI	528	1.0296
**YADVFHLYL**	877	0.0159	TITQMNLKY	538	1.6155	YIRKLHDEL	887	-0.2677
**RLSFKELLV**	365	0.8643	ISRQRLTKY	114	-0.6868	IERFVSLAI	856	0.1775
**HMLDMYSVM**	898	0.5853	LLKSIAATR	575	0.2055			
**VVDSYYSLL**	233	0.5381	RVCGVSAAR	10	0.6078			
**AMYTPHTVL**	923	0.1196	ATVVIGTSK	585	0.7566			
**SVAALTNNV**	397	0.3839	RAFDIYNDK	33	0.5993			
**MMILSDDAV**	755	-0.1616	TLKEILVTY	141	0.1118			
**KLLKSIAAT**	574	-0.4372	LTNDNTSRY	907	-0.3497			
**KLFDRYFKY**	281	-0.0533	ASGNLLLDK	383	-0.4206			
**RILGAGCFV**	836	0.3846	NLKYAISAK	543	1.3508			
**VTANVNALL**	700	0.1708	VAGFAKFLK	42	-0.7456			
**FMSEAKCWT**	793	0.3756	ALLSTDGNK	706	-0.1557			
			MLTNDNTSR	906	-0.3109			
			SQGLVASIK	772	0.6336			
			VVIGTSKFY	587	0.7146			
			LMPILTLTR	241	0.9617			
			RLSFKELLV	365	0.8643			
			VVSTGYHFR	341	1.4741			
			VVYRAFDIY	30	0.7331			
			DVFHLYLQY	879	0.6321			
			MVPHISRQR	110	0.4279			
			SICSTMTNR	561	1.1164			
			RIMASLVLA	631	0.1241			
			YIKWDLLKY	265	-0.321			
			KLHDELTGH	890	0.4147			

Supplementary Table S4. ToxinPred report for CTL epitopes having VaxiJen score above the threshold of 0.4

A2 supertype binding peptides			A3 supertype binding peptides			B7 supertype binding peptides		
**Amino acid sequence of the peptides**	**VaxiJen score**	**ToxinPred report**	**Amino acid sequence of the peptides**	**VaxiJen score**	**ToxinPred report**	**Amino acid sequence of the peptides**	**VaxiJen score**	**ToxinPred report**
**TMADLVYAL**	0.8208	Non-Toxin	HLYLQYIRK	0.492	Non-Toxin	FPPTSFGPL	1.6296	Non-Toxin
**NLIDSYFVV**	0.4282	Non-Toxin	KSAGFPFNK	1.1874	Non-Toxin	KPYIKWDLL	0.4825	Non-Toxin
**ILHCANFNV**	0.5385	Non-Toxin	TSFGPLVRK	1.7142	Non-Toxin	IPTITQMNL	0.7997	Non-Toxin
**MLDMYSVML**	0.5626	Non-Toxin	MTNRQFHQK	0.5341	Non-Toxin	ATRGATVVI	0.5688	Non-Toxin
**YTMADLVYA**	1.0142	Non-Toxin	IMASLVLAR	0.5587	Non-Toxin	TPCGTGTST	0.7838	Non-Toxin
**LLMPILTLT**	0.5803	Non-Toxin	LVASIKNFK	0.8148	Non-Toxin	CPAVAKHDF	1.1025	Non-Toxin
**SLSHRFYRL**	0.4779	Non-Toxin	LIDSYFVVK	0.4939	Non-Toxin	YLRKHFSMM	0.4887	Non-Toxin
**SLAIDAYPL**	0.7576	Non-Toxin	RVYANLGER	0.6654	Non-Toxin	MPILTLTRA	0.8061	Non-Toxin
**RQLLFVVEV**	0.816	Non-Toxin	MASLVLARK	1.3011	Non-Toxin	IAATRGATV	0.8883	Non-Toxin
**RLANECAQV**	0.9246	Non-Toxin	LSFKELLVY	0.7234	Non-Toxin	AALTNNVAF	0.6381	Non-Toxin
**KQGDDYVYL**	0.4041	Non-Toxin	TVKPGNFNK	1.3778	Non-Toxin	KPGNFNKDF	0.8932	Non-Toxin
**QLLFVVEVV**	0.821	Non-Toxin	AIDAYPLTK	0.5972	Non-Toxin	SAKNRARTV	0.9661	Non-Toxin
**SVLYYQNNV**	0.5524	Non-Toxin	CSLSHRFYR	0.629	Non-Toxin	FAYTKRNVI	1.0296	Non-Toxin
**RLSFKELLV**	0.8643	Non-Toxin	TITQMNLKY	1.6155	Non-Toxin			
**HMLDMYSVM**	0.5853	Non-Toxin	RVCGVSAAR	0.6078	Non-Toxin			
**VVDSYYSLL**	0.5381	Non-Toxin	ATVVIGTSK	0.7566	Non-Toxin			
			RAFDIYNDK	0.5993	Non-Toxin			
			NLKYAISAK	1.3508	Non-Toxin			
			SQGLVASIK	0.6336	Non-Toxin			
			VVIGTSKFY	0.7146	Non-Toxin			
			LMPILTLTR	0.9617	Non-Toxin			
			RLSFKELLV	0.8643	Non-Toxin			
			VVSTGYHFR	1.4741	Non-Toxin			
			VVYRAFDIY	0.7331	Non-Toxin			
			DVFHLYLQY	0.6321	Non-Toxin			
			MVPHISRQR	0.4279	Non-Toxin			
			SICSTMTNR	1.1164	Non-Toxin			
			KLHDELTGH	0.4147	Non-Toxin			

Supplementary Figures:

MRTSYLLLFTLCLLLSEMASGGNFLTGLGHRSDHYNCVSSGGQCLYSACPIFTKIQGTCYRGKAKCCK
EAAAKMLDMYSVMLAAYRQLLFVVEVAAYRLANECAQVAAYSVLYYQNNVAAYRLSFKELLVAA
YLSFKELLVYAAYTVKPGNFNKAAYRVCGVSAARAAYATVVIGTSKAAYNLKYAISAKAAYRLSFKEL
LVAAYVVSTGYHFRAAYKLHDELTGHAAYATRGATVVIAAYMPILTLTRAAAYIAATRGATVAAYA
ALTNNVAFAAYFAYTKRNVIGPGPGNMLRIMASLVLARKHGPGPGPNMLRIMASLVLARKGPGP
GQMNLKYAISAKNRARGPGPGMNLKYAISAKNRARTGPGPGNLKYAISAKNRARTVGPGPGDSY
YSLLMPILTLTRGPGPGMLRIMASLVLARKHTGPGPGLRIMASLVLARKHTTGPGPGLMPILTLTRA
LTAESGPGPGITQMNLKYAISAKNRKKAQDGNAAISDYDYYRYKKDKYFDCYDGGCINANQKKHF
RELGVVHNQDVNLH

Supplementary Figure S1. The designed vaccine construct sequence. The sequence of epitopes in the designed vaccine is: Adjuvant-CTL epitopes-HTL epitopes-B cell epitopes. The first 68 amino acids are occupied by the adjuvant, β-defensin 1 (Bue color), next 5 amino acids are of EAAAK linker ((Orange color) and the amino acids from 74-286 constitute the CTL epitopes (Red color) whereas the amino acids from 287-486 and from 487-540 correspond to HTL (Green color) and B-cell epitopes (Black color), respectively. All the linkers, except EAAAK are represented in purple color.

(A)

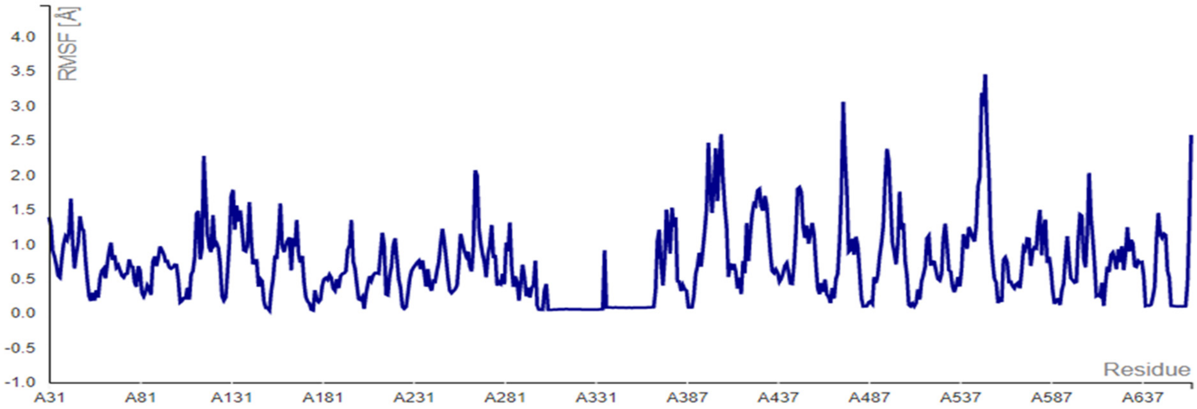

(B)

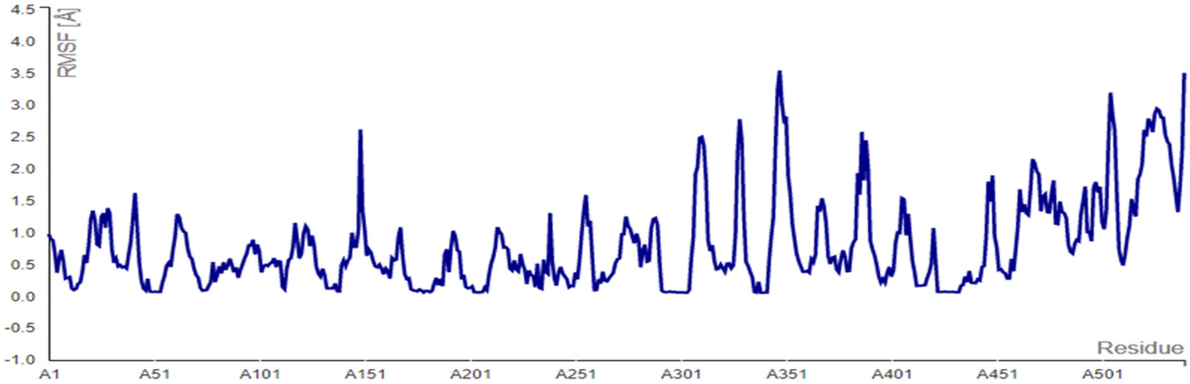

Supplementary Figure S2. Molecular dynamics simulations of the docked complex (TLR3-Designed vaccine). (A) The root-mean-square fluctuation (RMSF) values of the amino acid residues of Chain A (TLR3) of the docked complex. (B) RMSF values of the amino acid residues of Chain B (designed vaccine) of the docked complex.
